# Owner expectations and surprises of dog ownership experiences in the United Kingdom

**DOI:** 10.3389/fvets.2024.1331793

**Published:** 2024-02-07

**Authors:** Katharine L. Anderson, Katrina E. Holland, Rachel A. Casey, Ben Cooper, Robert M. Christley

**Affiliations:** Dogs Trust, London, United Kingdom

**Keywords:** dog, expectations, human-animal bond, pet ownership, dog acquisition

## Abstract

**Introduction:**

Although many owners are satisfied by dog ownership, large numbers of dogs are relinquished annually, with an estimated 130,000 dogs cared for each year by rescue organisations in the UK. Unrealistic ownership expectations are a potential factor in the decision to relinquish and therefore understanding what surprises owners about the realities of ownership and how this meets their expectations is vital.

**Methods:**

Using a retrospective cross-sectional cohort study design, as part of Dogs Trust’s National Dog Survey 2021, owners were asked ‘what has surprised you most about owning a dog?’ and to classify how their experiences had compared with their expectations on a list of aspects of ownership as either more than, less than or as expected. Free text responses (n= 2,000) were analysed using reflexive thematic analysis in NVivo Pro (v.12 QSR) and a quantitative summary of classified expectations (n=354,224) was conducted in R.

**Results:**

Many aspects of ownership were reported to be as expected, however a discrepancy between expectation and reality regarding some aspects was revealed. The cost of vet visits was greater than expected for the majority of respondents (52%), whilst other factors that often exceeded expectations included buying/rehoming cost (33%) and amount of patience needed (25%). Damage to furniture was less than expected for many (50%) as was damage to garden (33%). From the thematic analysis, four themes were generated that reflected what surprised owners most about ownership: emotional connectedness of human–dog relationships; dog’s impact on human health/wellbeing; understanding what dogs are like; and meeting the demands of ownership.

**Conclusion:**

Overall these results aid our understanding of dog-human interactions, highlighting the complexity of the dog-owner relationship which may come with unanticipated costs. Whilst this study’s results are reassuring given many aspects of ownership were as expected, and surprises were often positive, some areas had greater impacts than expected, raising opportunities for intervention, resources or support. The aim would be to manage owners’ expectations prior to acquisition or ensure these are more realistically met, reducing the likelihood of negative welfare implications for both dog and owner.

## 1 Introduction

Dogs are one of the most popular companion animal species across the world, including in the United Kingdom where dogs are owned by an estimated one-quarter of adults (1). Although many owners report satisfaction with their dogs and their relationships with them (2), significant numbers of dogs face negative welfare situations, such as relinquishment or euthanasia. Each year, in the UK, an estimated 130,000 dogs are cared for by rescue organisations (3). Factors associated with dog relinquishment are varied, including dog behaviour, owner ill-health, relocation or housing issues, lack of time, and financial costs (4, 5). Another factor associated with some cases of dog relinquishment is unrealistic or unmet owner expectations (4, 6). Unrealistic expectations for ownership were cited as a main reason for adopters returning dogs to shelters in 7 to 13% of cases (7, 8). Current evidence indicates a variety of dog- or ownership-related aspects associated with mismatched expectations. For instance, people adopting dogs from a UK charity who found the work and effort in looking after their dog to be more than they had expected had 9.9 times higher odds of giving their dog back to the shelter than people who found the effort required to be less than expected (9). Similarly, adopters returning their dog to the shelter within the first three months of adoption had significantly higher expectations for dog health and desirable behaviour, as well as the development of the human–dog bond, compared with non-returning owners (10). Excessive financial costs associated with dog ownership is another reported reason for relinquishment that suggests a discrepancy between expectations and reality (11).

Previous research has explored owner’s pre-acquisition expectations. In a recent survey of UK puppy purchasers, the misconception that some “designer crossbreeds” (e.g., Cocker Spaniel X Poodle, the “Cockapoo”) are “hypoallergenic” and thus pose a reduced risk to owner’s allergies was found to motivate owner demand for the purchase of such “designer crossbreeds” (12). This finding indicates potential risks to both dog and human welfare if owner expectations regarding a dog’s hypoallergenicity are not met. Other research suggests that owners of doodles (i.e., poodle hybrids, such as the “Cockapoo”) underestimated the maintenance and grooming needs of doodle dogs (13). Inadequate grooming can lead to potentially serious dog welfare consequences (14). In another survey, conducted in Australia, many prospective dog adopters anticipated health benefits of dog ownership to include increased walking (89%) and physical fitness (52%) (15). Respondents also expected improvements to mental health, through greater happiness (89%), and decreased stress (74%), loneliness (61%) and depression (57%). In the same study, dog training and dog behavioural issues were common expected challenges of dog ownership, anticipated by 62 and 50% of respondents, respectively. Expectations of dog ownership are shaped by experience and knowledge about dog behaviours and ownership requirements (16). Furthermore, evidence indicates that people with greater knowledge about animal care, health, behaviour, training and costs have more realistic expectations about dog ownership (e.g., the effort required) than people with less knowledge (17).

The relationship between the current perceptions of aspects of dog ownership experience and owner’s prior expectations has not yet been fully explored in a sample of current owners. Whilst there is an existing body of research about owners’ expectations surrounding dog ownership, there is a data gap regarding whether such expectations are perceived to be subsequently met. Furthermore, recent acquisitions during/since the COVID-19 pandemic has seen a potential increase to the pet dog population, with negative fallout possible particularly if dogs were acquired impulsively and/or with unrealistic expectations of ownership (18). Understanding which aspects of dog ownership surprise owners, and in what ways, is therefore a critical step in addressing dog relinquishment and optimising welfare for both humans and dogs. Using a sample of current UK dog owners, the objective of this study was therefore to retrospectively explore dog owners’ current perceptions of their expectations of dog ownership, reflecting on whether certain aspects of ownership were more, less or equal to what they expected. This study also investigated demographic/owner factor variables for their association with aspects that surprised respondents. Furthermore, the qualitative analysis aimed to broaden the scope of understanding of this topic, by identifying further aspects of dog ownership that surprised owners and produce richer insights into owners’ experiences.

## 2 Methods

Ethical approval for this study was granted by Dogs Trust Ethical Review Board (reference ERB050).

### 2.1 Data collection

This study used a retrospective cross-sectional cohort design, with an online survey used as the data collection tool. The “National Dog Survey 2021”, developed by Dogs Trust, collected responses from dog owners in the UK between 10th September to 25th October 2021. Full survey methodology including tool development, study participants and data collection has been previously described (19).

### 2.2 Data analysis

**Figure 1:**
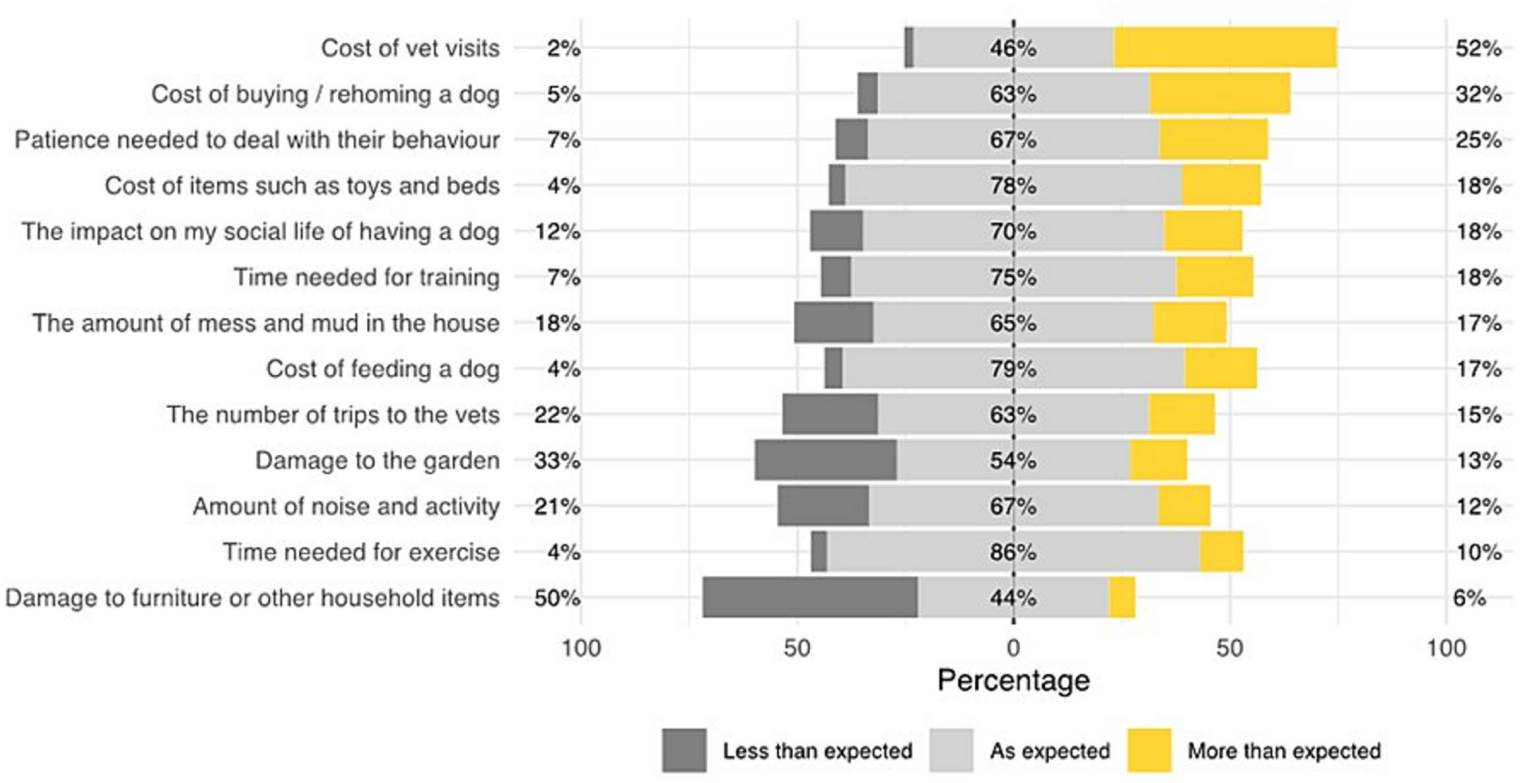
Percentage of respondents (*n* = 354,046) stating that their expectations of dog ownership (based on a series of 13 statements) were either less than, more than, or as expected.

To explore dog owners’ reflections on their expectations of dog ownership a convergent mixed-methods study design inspired the data analysis (20). Quantitative and qualitative data were collected in parallel, analysed independently and then interpreted together in a comparative and contrasting way. Data included within the qualitative analysis were responses to the free-text survey question “What has surprised you most about owning a dog?”, whilst the quantitative analysis summarises findings from the question “After owning a dog, please tell us which of the following are less, more or as you expected?” where respondents were asked to classify 13 functional areas of dog ownership (Figure 1) based on their experiences, as being “less than expected”, “as expected” or “more than expected”.

#### 2.2.1 Quantitative

Following the data cleaning methodology as described in Anderson et al. (19), data were imported into R (version 4.1.3) (21) and the distribution of the data checked. Descriptive statistics were then collated based on responses to the question “After owning a dog, please tell us which of the following are less, more or as you expected?”, and variables of interest were compared using the mean number of surprises (combining both more than and less than expected responses) cross tabulated to identify differences between groups. Variables of interest included age of the dog, due to the potential differences in experiences related to the current life stage of their dog, as well as number of dogs in the household as presence of multiple dogs may impact the comparison of expectations versus experience. Due to the previous literature highlighting that younger owners may be more likely to experience unrealistic expectations of dog ownership, owner age category was included within our analysis. Finally, acquisition factors such as source of acquisition and price paid for dog were included due to potential differences in owners’ expectations when acquiring their dog through different sources. Statistical comparisons were considered inappropriate due to the large sample size, increasing the likelihood of introduction of false positive results.

#### 2.2.2 Qualitative

From the full dataset where answers were provided to the question “What has surprised you most about owning a dog?” (*n* = 273,899), a random subsample of 2,000 free-text responses was generated in R using slice_sample() from all comments that were not blank and contained at least 3 characters. These responses were imported into NVivo Pro (v.12 QSR) and analysed following a reflexive thematic analysis approach (22, 23). The research question that guided the qualitative analysis was “What aspects of dogs or ownership surprised owners since their dog’s acquisition?” This closely resembled the free-text question from which the data were obtained. An experiential orientation to data interpretation was adopted as we aimed to prioritise owners’ own accounts of their experiences and perspectives. One author (KH) familiarised herself with the data by reading all 2,000 free-text responses, generating initial codes from the data and then organising them into meaningful groups from which themes were constructed. Data were inductively coded, in that coding was driven by the content of the responses, rather than pre-determined codes. As coding progressed, some codes were modified. From the initial coding, categories were identified which were then collated into themes. The themes linked ideas and concepts within grouped codes that represented overall patterns of meaning that the researcher interpreted from the data. Following the same process, another author (KA) independently coded 1,000 responses from the subsample. This was done not with the goal of achieving greater reliability or accuracy between the coders, but rather to deepen our reflexive engagement with the data, for instance by identifying any overlooked areas in our respective analyses. Three authors (KH, KA, and RMC) met to review KH and KA’s construction of themes and relevant data references, and collaboratively established the final themes.

Whilst an inductive approach was employed for coding and theme development, we recognize that the qualitative researcher always, unavoidably, brings their pre-existing knowledge to the analysis. As researchers in the field of dog welfare research, both coders (and the wider research team) were familiar with prior research on the topic being explored. Most authors also had dog ownership experience. We acknowledge that the team’s pre-existing knowledge and experience may have informed our understanding of respondents’ experiences and the aspects they found surprising.

Including the full dataset within the qualitative analysis was neither feasible, for manual coding, nor necessary, to meet the goal of this study’s qualitative aspect. In line with research conducted within a qualitative paradigm, the aim of this study’s qualitative analysis was to explore some of the range and diversity of experience amongst dog owners, rather than present a quantified representation of the data. Through engagement with the subsample outlined above, including appraisal of the breadth of the study’s aim and the richness of the individual data items, the researchers determined that this subsample size had adequate “information power” (24) to meet this study’s aim.

## 3 Results

A total of 354,046 respondents owning dogs in the UK completed the survey. Respondents were asked to complete one survey per household and for their most recently acquired dog. Full demographic summaries of respondents are available in a previous publication (19).

### 3.1 Quantitative analysis

Respondents were asked to classify a series of statements based on their current perception of ownership as either as “expected”, “less than expected” or “more than expected”, with the overall results of this study revealing insights in areas of ownership, indicating several aspects of ownership in which owners reported that their expectations were commonly under- or over-met (Figure 1). The greatest discrepancies where expectations were exceeded were the cost of vet visits (52% of respondents), cost of buying/rehoming a dog (32%) and patience needed to deal with behaviour (25%). Areas that were often less than what was expected included damage to furniture and other items (50%) and damage to garden (33%).

When considering the number of surprises experienced by respondents, both “more than expected” and “less than expected”, the mean number of surprises reported decreased steadily with an increase in owner age category. Respondents in younger age categories experienced a greater number of surprises, while older owners more frequently reported ownership aspects were as expected (Table 1). Younger respondents more frequently reported surprises around: amount of noise and activity, cost of feeding, and toys/beds, damage to furniture, social life impacts, patience needed to deal with behaviour, amount of mess, and time needed for exercise and training. One exception was the cost of vet visits, which was reported to be “more than expected” across all age groups; furthermore, the “more than expected” surprise at cost of vet visits also was more frequently reported as dog age increased.

**Table 1:** The mean number of surprises overall, and less than or more than, when classifying 13 statements about aspects of dog ownership as more than, less than or as expected.

		Mean number of surprises		
Variable	Number of respondents	Less than expected	More than expected	All surprises (both more than and less than expected)
Owner age group (years)				
18–24	17,423	2.64	3.27	5.92
25–34	46,273	2.22	3.05	5.27
35–44	52,775	1.78	2.70	4.48
45–54	96,723	1.69	2.45	4.14
55–64	90,272	1.68	2.31	3.99
65–74	43,450	1.84	2.22	4.06
75 or over	8,337	2.18	2.00	4.18
Number of dogs owned				
1	256,892	2.09	2.56	4.65
2	96,727	1.44	2.48	3.92
3	19,188	1.26	2.34	3.61
4	5,713	1.24	2.33	3.58
5+	3,446	1.16	2.11	3.27
Age of dog (grouped by years)				
0	36,417	1.71	3.02	4.74
1	42,294	1.82	3.06	4.88
2	33,529	1.79	2.72	4.51
3	33,417	1.85	2.55	4.40
4	31,046	1.88	2.47	4.35
5	29,453	1.88	2.41	4.29
6	27,719	1.89	2.36	4.24
7	26,658	1.92	2.28	4.20
8	25,259	1.92	2.29	4.21
9+	96,257	1.90	2.26	4.16
Price paid for dog				
No cost/gift	49,460	1.95	2.32	4.27
Up to £100	27,398	1.98	2.18	4.15
£100–250	75,169	1.96	2.25	4.21
£251–500	79,672	1.83	2.47	4.30
£501–1,000	87,614	1.76	2.65	4.41
£1,001–2000	45,872	1.77	2.98	4.75
£2001–3,000	14,447	1.83	3.31	5.13
Over £3,000	2,417	1.97	3.30	5.27
Source of acquisition				
Local press	6,618	1.76	2.34	4.10
Rehoming website	47,172	1.95	2.14	4.10
Rehoming visit	16,763	2.13	2.15	4.29
Breed group website	10,033	1.66	2.51	4.18
Breeder visit	21,799	1.82	2.47	4.30
Breeder website	13,131	1.82	2.61	4.42
Local community	8,982	1.84	2.46	4.30
Local adverts	2,024	1.91	2.40	4.31
Kennel club website	23,885	1.72	2.60	4.32
Foreign rehoming website	10,559	1.92	2.45	4.37
Family and/or Friends	68,926	1.91	2.50	4.40
General website	31,484	1.83	2.71	4.54
Social media	17,088	1.94	2.63	4.57
Pet website	79,786	1.80	2.86	4.66

A higher number of mean surprises was recorded in those who owned fewer and younger dogs (Table 1). Those with fewer dogs also more frequently reported that damage to garden and furniture was “less than expected”. Owners of younger (particularly 0–2 years) dogs more frequently reported that damage to garden, amount of mess, time needed for training and patience needed were “more than expected” compared to owners with older dogs. The mean number of surprises was also lower for owners who acquired their dogs from rehoming centres, and higher in those getting dogs from general selling or pet selling websites. Those who acquired their dogs from rehoming centres also reported higher frequency of “less than expected” than other sources.

Finally, those who paid more for their dogs had a higher mean number of surprises and reported higher numbers of “more than expected” within their responses (Table 1). The amount of patience needed to deal with their dog’s behaviour was reported as “more than expected” more frequently by owners that had acquired their dog from foreign rehoming websites or pet selling websites. Where owners had acquired their dogs from websites, both pet and general sites, higher frequencies of “more than expected” were reported by owners for: more noise and activity, damage to furniture and garden, amount of mess and mud in the house, and time needed for training compared to other sources. Finally, owners sourcing their dogs from Kennel Club websites reported number of vet visits to be “more than expected” more frequently than owners who acquired their dogs from any other source.

### 3.2 Qualitative analysis

Of the codes generated from the responses, four distinct but interlinking themes were constructed: Emotional Connectedness of Human–Dog Relationships; Dog’s Impact on Human Health or Wellbeing; Meeting the Demands of Dog-Ownership, and; Understanding What Dogs are Like. Together, these themes reflect both emotional and practical dimensions of dog ownership and, overall, illustrate that dogs occupied a more prominent place in their owners’ lives than they had anticipated. The themes are outlined in Table 2. Data excerpts contained in Table 2 are referenced in the written account below, to illustrate the themes. In addition to these themes, some people commented that nothing had surprised them. These respondents often linked their lack of surprise to their previous ownership experience (e.g., “I have always had dogs so no surprises really.”), suggesting that owners’ expectations are shaped, in part, by their dog ownership history.

**Table 2:** Key themes from the thematic analysis relating to surprises associated with dog ownership when asked “What has surprised you most about owning a dog?”

Themes and definitions	Sub-themes		Example data extracts
1. *Emotional connectedness of human-dog relationships*: owners were surprised by the quality and depth of relationships formed, and interactions experienced, between themselves and their dog	Companionship dog provides		*“How much I value her company.”* (1a)
	Friendship between human and dog		*“How I truly consider him to be one of my best friends*.” (1b)
	Dogs as part of the family		*“How much of a vital role in the family they play.”* (1c) “*How attached you get, they become your children.”* (1d) *“[Dog name] is the most demanding yet affectionate dog I’ve ever owned, he’s like a child.*” (1e)
	Love and affection between human and dog		*“I never imagined that I could love the way I love [dog name]*.” (1f) “*How much love and affection they have to show you*.” (1 g) *“The love they give is pure and unconditional.”* (1 h)
	Ease or speed of relationship forming		*“How quickly you fall in love with him and how quickly he becomes part of the family.”* (1i)
	Attachment between human and dog		“*I never in a million years realised how attached I would get to a dog. I’ve owned cats before but never had the emotional attachment like this.”* (1j) “*How attached ive [sic] got to him, could not imagine not having him.”* (1 k) *“The unbreakable bond between me and my dog.”* (1 l)
2. *Dog’s impact on human health or wellbeing*: owners were surprised by the effect their dog, or dog ownership, has had on their health or wellbeing	Dog improves health or wellbeing	Dog makes owner feel good	“*I got more happiness than what I thought I would get, seeing him gain confidence and learn is very rewarding.”* (2a) *“How calm I feel and the enjoyment I get from a long walk.”* (2b) “*How much calmer I am just being with them cuddles on the sofa, or just sitting together helps my anxiety.”* (2c) *“How I do not feel lonely anymore.”* (2d) *“[G]ive me a reason to get up even on the toughest days.”* (2e)
		Mental health conditions improved through dog	*“The way it changed the whole family he helped my daughter immensely as she suffers with anxiety and depression.”* (2f)
		Emotional support or comfort dog provides	*“How much [dog name] has comforted me when my parents passed away.”* (2 g) *“The impact he has on everyone who spends time with him. We have a friend who asked to spend time with [dog name] as it helped him through a difficult time.”* (2 h)
		Social interactions or connections through dog	*“The friends I have made from walking the dogs.”* (2i) *“[T]alking to strangers because they do not treat you like your [sic] odd when you have got a dog with you and you talk to them.”* (2j) “*How he has united our family.*” (2 k)
		Physical health has benefitted	“*[I]mproving my fitness* via *walking.”* (2 l)
	Dog puts a strain on health or wellbeing	Worries about their dog	*“The emotional drain of worrying about how to help my dog best and what more could I be doing to help her.”* (2 m) “*How much I would fall in love with him, but also how much then I worry about him too.”* (2n)
		Heartbreak when they pass away	*“How devastating it is to lose them its [sic] like losing family*.” (2o)
		Emotional strain of dog’s behaviour or training	*“As our second dog [dog name] has been hard work compared to our previous boy to the point of considering rehoming him. Been soul destroying at times.”* (2p)
3. *Meeting the demands of dog-ownership*: owners were surprised by the amount of time, work, effort or money involved in caring for their dog, and how meeting their dog’s needs affects their everyday life	Dog ownership is time consuming		*“The amount of time required to spend with your dog e.g. [sic] Walking, playing, training* etc.” (3a) *“Having only had one dog before, a fourteen year old male mixed breed, and gone into owning a puppy with my eyes open, I have still been surprised at how totally full on she is, needing to be watched every waking minute, in or out of the house! Also, If it’s within reach, it will be in her mouth.*” (3b) “*How difficult puppies are to look after and how much constant training they need*.” (3c)
	Dog ownership can be hard work		“*Puppy training is very hard work when the puppy has not got an older dog to learn from.*” (3d) “*[H]ow challenging it can be to have a rescue dog.*” (3e) “*How much work it is but how much you get out of it.*” (3f)
	Dog ownership is expensive		“*Owners since 1989 […] Vets have gone very greedy*.” (3 g) “*The behaviour (selling up techniques) of vets, e.g. Routine drip following minor surgery plus pre-op blood tests for puppy tooth extraction and stitches.”* (3 h) *“The unexpected costs of vet fees and how everything seems to be a £100 minimum for even just a simple ear infection.*” (3i) “*Worst thing insurance is a nightmare especially when your [sic] dog is getting old.*” (3j)
	Commitment and responsibility towards dog		“*[T]he commitment as we cannot leave him.*” (3 k) “*How much responsibility I feel towards his happiness.*” (3 l) “*The responsibility to train and teach.*” (3 m)
	Everyday life organised around dog		“*It is very tying and doing normal things like going out or arranging holidays need much more thought.”* (3n)
	Society is not always dog-friendly		“*[H]ow many places aren’t dog friendly.*” (3o)
4. *Understanding what dogs are like*: owners were surprised by what dogs are like, including aspects of dog’s temperament, behaviour or abilities	Dogs’ personality or temperament		*“His personality—it’s HIUUGE!” [sic]* (4a) “*How loyal and loving they are.”* (4b) *“I have had dogs all my life but never had a Labrador it delights me that how friendly our dog is.” [sic]* (4c) *“[H]ow much fun they are.”* (4d) *“I have had many dogs but this one is so good she has never chewed or eaten anything she is not allowed.”* (4e) *“The only thing that surprised me is the different temperaments of different breeds and I love how each dog has its own unique personality.”* (4f)
	Dog’s energy or activity levels		“*This has been the most energetic puppy I have ever owned. My last doodle was very calm.”* (4 g) *“How much she sleeps during the day!!”* (4 h)
	Dogs’ intelligence		“*[Dog name] is different from all the others iv [sic] had, in that in human terms, I think he borders on being a genius, i cant [sic] believe how easy to train he was.*” (4i)
	Communication between humans and dogs		“*How astute dogs are. They know when you are upset or need reassurance and give it to you (all mine have).*” (4j)
	Dog’s ability to transform		“*Considering [dog name]’s rescue background when we rehomed her at 4 months old how well she has adapted to life with us and how lovely and trusting she is with people and other dogs.*” (4 k)
	Dog’s behavioural problems		*“He’s a sweet dog with a lovely personality, but his barking can upset people and other dogs.*” (4 l) *“Howling when I leave him even for short periods. My previous dog had no problem being left alone.*” (4 m) *“The initial puppy teething stage was a real big shock!*” (4n) *“The 6 month old (adolescent) regressive behaviour. He seems to be pushing boundaries and forgetting all that he’s learnt with training.*” (4o)
	Dogs are dirty or messy		“*How much bloomin [sic] hair this dog has and leaves everywhere on my carpet.”* (4p) “*How much he poos and how bad his trumps smell.*” (4q)

#### 3.2.1 Emotional connectedness of human-dog relationships

This theme encapsulates respondents’ surprise regarding the intimacy of relationships established between themselves and their dogs. One valued aspect of human-dog relationships, that surprised some owners, was the company dogs provide (1a). Beyond occupying a purely functional role in owners’ lives, however, many respondents alluded to deeper human–dog relationships, commenting that they enjoyed friendship with their dog (1b), or considered their dog to be deeply embedded in the family unit (1c). Emphasising the emotional bond they share with their dogs, some respondents described the relationship with their dog as though the dog was akin to a child (1d; 1e). Many owners expressed surprise regarding the amount or depth of love and/or affection that they felt towards their dog and/or that they received from their dog (1f; 1 g). The love that dogs give their owner was often valued for its “unconditional” quality (1 h). Owners emphasised a few types of close relationships with their dogs (e.g., friendship or family) that were often described as developing quickly (1i). Many owners described feeling more attached to their dog than expected (1j), with some commenting that they miss them when they are not together or that they cannot now imagine life without their dog (or a dog) (1 k). Whilst some respondents focused on the attachment they felt to their dog, others noted that this feeling was reciprocal as they described a strong mutual bond between themselves and their dog (1 l).

#### 3.2.2 Dogs’ impact on human health or wellbeing

This theme encapsulates participants’ surprise regarding the impact that their dog – or aspects of dog ownership – was perceived to have had on the health or wellbeing of themselves or other people, most typically a family or household member. Most references to health or wellbeing highlighted positive perceived changes to psychosocial aspects of health (i.e., mental, emotional, and social). Many respondents referred to improvements, since dog acquisition, in areas of mental health, including intrinsic positive feelings of happiness or enjoyment (2a; 2b). Sometimes these outcomes were associated with the time owners spent with their dog, including through specific activities, such as walking (2b), or as a result of the affection and love dogs give their owner (2c). Here, this theme connects to the theme *Emotional Connectedness of Human-Dog Relationships*. For some owners, dogs were felt to increase feelings of ease (2c), mitigate loneliness (2d), and provide purpose or motivation to get up or go outside (2e). Some respondents noted specific mental health conditions that they felt their dog had helped to alleviate (2c; 2f). Responses suggested that, in some cases, the perceived improvements to mental health may have been mediated (at least partially) by the emotional support or comfort that dogs were widely reported to provide, particularly during difficult periods in a person’s life (2 g; 2 h). Respondents were also surprised by the increase in social interactions they had experienced as a dog owner, sometimes forming social connections with other people through walking their dog (2i). Dogs were described as acting as a catalyst for conversation with strangers who they would not otherwise interact with (2j). As well as interactions and connections with strangers, some participants commented that their family dynamic had improved since acquiring their dog, with the dog perceived to have brought family members together (2 k). In addition to the emphasis that many respondents placed on impacts on psychosocial health, some respondents also highlighted benefits to physical health, achieved through increased exercise via walking (2 l).

However, some respondents expressed surprise at how they perceived dog ownership to have compromised their wellbeing. Worries about meeting their dog’s needs through optimising their health and happiness were reported (2 m), with some suggesting that their concerns were associated with their close relationship with, or attachment to, their dog (2n). Respondents also related their attachment to their dog with the emotional distress owners experience as dogs age and die (2o). This aspect of this theme should thus be interpreted in conjunction with the theme *Emotional Connectedness of Human–Dog Relationships*. A further threat to owner’s wellbeing was the emotional strain associated with the often-reported “hard work” involved in raising a puppy or owning a dog with challenging behaviour (2p), which was an aspect of the theme *Meeting the Demands of Dog* Ownership.

#### 3.2.3 Meeting the demands of dog ownership

This theme is characterised by respondents’ surprise regarding the extent of dogs’ needs and how owners meet these. Meeting the demands of ownership was described by respondents in two primary ways: (1) through the provision of largely tangible things, for instance time (i.e., time spent training or exercising) or money, and; (2) how fulfilling their dog’s needs was associated with an all-encompassing caregiving role performed by the owner. Together, these aspects reflect the practical and affective dimensions of meeting the demands of dog ownership.

The first aspect of this theme concerned owners’ surprise regarding the extent of owner involvement or the amount of resources required to fulfil a dog’s needs. Some respondents emphasised their surprise regarding the amount of time and attention dog ownership involved (3a; 3b). For instance, owners noted that the amount of time required to meet their dog’s training needs was greater than expected, with some suggesting that they have found training to be an ongoing process, rather than an activity that can be fully completed (3c). For some respondents, dog ownership had been greater or harder work than anticipated, often due to the amount of training or care required (3d). An emphasis on more hard work was particularly expressed by owners of puppies (3d) or rescue dogs (3e), with these owners associating the hard work with inherent challenges they perceive to accompany these types of dogs. However, several respondents expressed that the hard work was worth it, given how rewarding they found dog ownership to be (3f). As well as the demand on owner’s time and effort, some respondents had not expected the financial costs associated with dog ownership to be so high. Veterinary costs were a commonly reported surprising expense. Some owners perceived veterinarians to be greedy (3 g), for instance by “upselling” procedures (3 h) or charging more than owners anticipated for issues they considered to be routine or minor (3i). The cost of insurance was also reported as surprising by a minority of owners, with some placing emphasis on the rising cost of insurance as dogs age (3j).

In attempting to fulfil their dog’s needs, some participants emphasised the level of commitment (3 k) or responsibility that they found to be required of dog owners, or that they felt towards their dog. A few respondents explicitly associated their sense of responsibility with their desire to optimise their dog’s happiness (3 l) or meet their training needs (3 m). Owners’ commitment to their dog’s wellbeing led them to perceive their lives as being organised around their dog’s needs. One consequence of this was a sense of restriction around owners’ spontaneity, with some viewing their dog as a tie – for instance, limiting their ability to travel (3n). A minority of respondents also noted their surprise that society – particularly hospitality businesses – is not always welcoming to dogs, adding to the additional forward planning required when wanting to go out and about with their dogs (3o).

#### 3.2.4 Understanding what dogs are like

This theme encapsulates elements that comprise how dogs act, or who they are perceived to be as individuals, primarily in terms of their temperament, abilities and behaviour. One aspect that commonly surprised respondents was the amount of personality or character their dog has (4a). Specific personality traits noted were predominantly positive, including loyalty (4b), lovingness (4b), sociability or friendliness (4c), and fun (4d). Some respondents were surprised by how well-behaved their dog is (4e). There was an emphasis on dogs’ individuality as an aspect that surprised people, with some commenting on differences between different dogs’ temperaments (4c; 4e; 4f). As well as the individuality of dogs, some respondents reported their surprise regarding perceived breed-based differences in temperament (4f). A further aspect that surprised some owners was their dog’s activity or energy levels. A few people with previous ownership experience commented on the difference between energy levels in their current and formerly owned dogs (4 g). A small minority of respondents were surprised by the amount of time their dog spends sleeping (4 h).

Some respondents were surprised by how intelligent they perceived their dog to be, sometimes inferred from their dog’s ease of learning (4i). As well as general intelligence, dogs’ communicative abilities with people were also noted, with some owners commenting on their dog’s remarkable ability to understand and respond to human emotions and moods (4j).

A dog’s ability to adapt to their new surroundings surprised some respondents, particularly those who had adopted their dog (e.g., acquired from a rehoming organization). Some were surprised by how well or quickly their dog had settled in (4 k).

However, some aspects of dog temperament or behaviour were less positively regarded, as some respondents reported being surprised by elements of their dog’s behaviour they considered problematic. A range of specific issues were noted, including barking, which a minority of participants reported was a problem either for themselves or others (4 l). Separation-related behaviours (e.g., crying when left home alone) surprised a few others (4 m). A minority of respondents commented on behavioural issues associated with puppies, including puppy teething or biting (4n) and behavioural regression during adolescence (4o). Dog’s individuality was highlighted in some owners’ accounts of behavioural issues, whereby individual dogs had been more or less challenging than other dogs owned by the respondent (4 m).

A minority of participants were surprised by the messy and dirty aspects of their dog or dog ownership. The amount of hair that dogs shed was a commonly reported issue, with some respondents describing dog hair as ubiquitous in the home (4p). Some respondents reported aversion around their dog’s bodily functions, for instance referring to the amount of poo the dog produces (4q).

## 4 Discussion

The aim of this study was to explore how the experience of dog ownership compared with owner expectations in a sample of UK dog owners. Overall, the results of this study provide initial insights into certain areas of expectations surrounding dog ownership where owners may experience discrepancies and surprises related to dog ownership. The findings highlight several aspects of dog ownership that surprised owners in both positive and negative ways, which can be utilised to guide future research in this area as well as develop interventions aimed at supporting dog owners to reduce the likelihood of negative fallout, such as relinquishments, due to these discrepancies.

One key area of surprise for owners within this study was around meeting the demands of dog ownership, which encompassed both the extent of dogs’ needs and the capability of owners to meet these. This was identified through both the qualitative thematic analysis and the quantitative analysis of graded statements. Within this theme, it was common for respondents to be surprised by factors related to costs associated with dog ownership. Common findings within this study were unexpected costs relating to the care of the dog, such as feeding them, providing necessities such as beds and toys, and keeping them healthy. Previous research has also demonstrated that unexpected or excessive financial costs are perceived as a challenge in dog ownership (15). Within the quantitative data, the greatest surprise reported by owners was the costs related to veterinary care, with owners reporting this more frequently as the age of the dog increased, suggesting unmet expectations can occur some distance in time from acquisition. This increase in surprise of vet costs in owners of older dogs may either reflect a perceived increase in the costs associated with health conditions particularly prevalent in older dogs, or a wider perceived increase in service costs over time. This was also a key sub-theme within the qualitative analysis where many were surprised at the cost of care, particularly things they themselves considered minor or routine. This suggests that some owners may perceive veterinary surgeries to be overcharging or profiteering, when in reality many veterinary procedures, including routine ones, are cost intensive. With the UK’s funded public health system, owners may be unlikely to compare veterinary healthcare costs to that of a human healthcare setting, raising the question as to how owners benchmark veterinary costs. Research from the United States suggests that pet owners and veterinarians may differ in how they relate to veterinary costs (25). When discussing the costs of veterinary care in focus groups, pet owners suggested that animal care should come before profit, while veterinarians focused on the tangible aspects of their services (e.g., time) and felt that their work is undervalued. While some existing research in other countries has explored public perceptions of the veterinary profession (26), there is a lack of evidence pertaining to UK pet owners’ perceptions of the veterinary profession. Further research into public perception of the veterinary profession and the veterinarian-client relationship in the UK would be beneficial to understand this further, as well as to better understand cost-related barriers to obtaining veterinary care and ways to mitigate these. Breed can also impact on expectations of costs, with owners of brachycephalic breeds more prone to underestimate the veterinary cost of their dog due to the high disease burden seen in these breeds (27). Financial burden may occur where expectations of costs are exceeded. With financial reasons often reported as factors associated with the relinquishment of pets (28–30) and in particular cats and dogs, it is important that owners are aware of and understand the potential lifetime financial costs, particularly routine costs, involved in dog ownership prior to acquisition.

Successful human-dog relationships often rely on met expectations, with ownership offering a symbiotic relationship for both dogs and their owners. As part of this relationship, it is imperative that owners invest the necessary time to provide their dogs with what they need, for both their dog’s welfare and their own, with research highlighting owners may experience negative wellbeing due to feeling they had not met their dog’s needs or expectations (31). Within this study, another area of surprise was the amount of time and effort needed to provide the necessary care for a dog. Often owners felt like their lives were organised around their dogs to fulfil their dog’s needs, and the level of commitment and responsibility was often noted as a surprise. Misconceptions of the amount of time needed for elements such as exercise and training may exist, particularly to those who may not conduct sufficient research before acquiring their dog or acquire a dog ill-suited to their lifestyle. A common owner-related reason for relinquishment of dogs is due to lack of time to spend with the dog, not being able to provide the time they need (15, 32–34), whilst studies also highlight the increase in responsibility as a challenge of dog ownership (15, 27, 33). Scarlett et al. (34) found this was most common for owners of younger (less than 2 years) dogs, with 70% of owners having owned their dog less than a year, suggesting that in many cases this may be due to unrealistic expectations of time requirements, rather than changes of circumstances. Misconceptions of what specific breeds need has also been highlighted, with a study highlighting that owners of brachycephalic breeds were commonly surprised at the level of exercise and maintenance their dogs required (27).

Training is an important element of dog ownership and, within this study, the amount dogs needed often exceeded their owners’ expectations and there was surprise that this was a continual ongoing process and need throughout the dog’s entire life. Problematic behaviours are common reasons for relinquishment, posing significant concern and challenges to dog owners (15, 17, 31, 33, 34) and without adequate training and appropriate behaviour modification, subsequent undesirable behaviours may develop. Some aspects of dog behaviour were reported negatively by respondents with many owners being surprised by their dog’s problematic behaviour. This included issues such as separation-related behaviour and barking. Previous research has highlighted that dogs that behaved in unexpected ways resulted in reduced emotional closeness and attachment to dog – highlighting potential damage to the human-animal bond if expectations are not met (27). Furthermore, many owners were surprised by the amount of attention their dog needed, as well as the amount of patience; this was particularly true for those owning puppies and rescue dogs. While this unmet expectation has the potential to jeopardise the human-animal bond, for many the hard work was however worth it due to the rewards of ownership.

Another key finding of this study was the surprise that dog owners had around understanding what their dogs are like. When asked to construct their “ideal” dog, in a study by King et al. (35), owners reported a number of physical and behavioural traits that were important to them. This included traits such as friendliness and obedience, with women preferring traits such as calm and sociable and men selecting traits such as energetic and faithful. Evidence further suggests owners acquire dogs to provide a source of companionship, and therefore likely select a breed reported to offer a suitable personality and temperament to match their expectations (15, 36, 37). Despite this, within our research many respondents were surprised by elements of the way their dogs were with regards to their individual personalities and temperament, how much character they had, as well as their levels of activity and energy. It was also common for owners to report surprises when comparing their current dog to previously owned dogs, such as differences in temperament and energy levels. Research has shown owners may be more likely to return/relinquish a dog when comparing them to a previously owned dog’s needs and traits, due to being less tolerant of what they may consider misbehaviour (15). Dog needs and behaviour can both vary greatly between and within breeds (38, 39), and therefore expecting dogs to behave similarly to previously owned dogs, particularly if the same breed, may result in unrealistic expectations and ultimately negative outcomes.

The qualitative analysis provided deeper insight into factors not captured by the quantitative data in this study, such as the emotional connectedness of the dog-owner relationship, which was often reported as a surprise by owners. Existing literature suggests that companionship is a commonly expected benefit of dog ownership (15), with numerous studies reporting that companionship for the owner is a primary motivation for dog acquisition (37, 40–42). Companionship for others in the household (including children, adults or dogs) is another common reason for getting a dog (37). Therefore, this study’s qualitative findings indicate that the perceived amount and depth of companionship provided by dogs may be even greater than expected. Similarly, whilst previous research has found that dogs are often understood as family members (36, 43–49) and that, in Britain, pets have been considered as friends since the end of the 17th century (50), the closeness of owner-dog relationships nevertheless surprised many of our respondents.

Consistent with previous literature (47, 51) respondents associated strong human-dog attachments with the emotional support or comfort they perceived their dogs as providing them with. Whilst evidence indicates that prospective owners often anticipate mental and physical benefits (15, 36), many respondents in this study reported these as taking them by surprise, suggesting that this is another aspect of ownership that may be difficult to fully comprehend prior to acquisition. Despite owners’ surprise regarding the emotional benefits of dogs or dog ownership, this study’s qualitative findings also highlighted some respondents’ surprise around the more unpleasant emotions associated with dog ownership. Feelings of worry and guilt about whether they were sufficiently meeting their dog’s needs took some owners by surprise. This perceived negative impact of ownership on owner wellbeing has been similarly identified in previous research (51). Perceptions of pets as a source of worry were highlighted in studies of pet ownership experiences during the COVID-19 pandemic, with owner’s worries often linked to restricted access to veterinary care, food supply chain issues, or exercise restrictions during this time (52–54). Beyond everyday worries, owners in this study also reflected on their surprise regarding the emotional distress felt when dogs die. Some respondents’ comments suggest that greater attachment with a dog may be positively associated with the severity of grief experienced or anticipated when dogs die, which echoes findings from previous research (51, 55–58). Consistent with the contradictory findings in existing literature (51) our research suggests both positive and negative perceived impacts of dog ownership on owner’s wellbeing exist. Overall impacts of dog ownership (whether positive and/or negative) on owner wellbeing is likely dependent on the individual dog, owner and their unique relationship (51).

This study’s findings showed that many aspects of dog ownership were reported as surprising. Evidence suggests that around half of dog owners carry out pre-acquisition research ahead of acquiring their dog (59). It is therefore perhaps unsurprising that many owners report elements of ownership as not what they expected. In those that do conduct pre-acquisition research (and from appropriate sources), this disparity in their expectations may be due to prospective owners having difficulty in fully comprehending the lived reality prior to acquisition. In order to address this, interventions could include physical preparations such as spending time caring for dogs, e.g., co-care of a friend’s dog/fostering, on top of the desk-based research owners are encouraged to conduct before acquiring their dogs could, in order to prepare owners for the realities of dog ownership. Further research into expectations around dog ownership is warranted, as well as assessing interventions aimed at encouraging pre-acquisition research. Preparing owners ahead of acquisition of a dog may well aid understanding around the process of acquiring a dog, and reduce the likelihood of negative fallout such as decreased welfare, damage to the human-animal bond and relinquishment or euthanasia.

## 5 Limitations

This study indicates a number of areas that owners may experience discrepancies in expectations versus experience regarding dog ownership, however this study’s findings are subject to several limitations. Firstly, a lack of information regarding respondents’ expectations before acquisition precludes us from making direct comparisons between pre- and post-acquisition expectations and experiences. For example, we do not know how cautious or optimistic they were. Our data also did not capture respondents’ previous dog ownership experience, with previous dog experience shown to be an influential factor on owner expectations (16); this limits the conclusions drawn from the data somewhat. Secondly, our data are cross-sectional, and we expect that respondents would have owned their dogs for varying lengths of time. The length of time the dog had been owned could have affected respondents’ perceptions and may also introduce recall bias relying on respondents to recall information from a long time ago. For instance, previous research has found that dog adopters’ expectations for ownership relative to their experience changes over time: owning a dog was considered easier than they had expected by an increasing proportion of adopters over time (10). Finally, the population of dogs within this study could be considered “successful” relationships, given they are still living with their owner, suggesting that many owners whose experience violates their expectations may simply manage this. However due to limited questions in our survey regarding welfare, we are unable to comment on the impacts of mismanaged expectations on the welfare or both owner and/or dogs, nor can we predict subsequent outcomes from this study, such as relinquishment or euthanasia. Further research in this area would benefit from longitudinal study design exploring owners expectations both pre- and post-acquisition, collecting additional information such as that listed above within the limitations of this research to allow for a more thorough understanding of the discrepancy between expectations and actual lived reality. Furthermore, understanding how expectation discrepancies and surprises of ownership have impacted dog owners and outcomes such as relinquishment is warranted to provide deeper insight in this area.

## 6 Conclusion

Dog ownership can be very valuable to humans; therefore, it is unsurprising that they are most commonly owned companion animal in many countries, including the UK. Often attention is drawn towards the perks of ownership, and the realities and consequences therefore may not be widely considered by potential owners. Overall, this study indicates that owners’ reflections on dog ownership are complex and that this type of human-animal relationship involves forming close relationships often at a greater cost (e.g., financial and time) than anticipated. This study highlights that particular aspects of dog ownership, such as the strength of bonds and the extent of the benefits and challenges, may also be difficult to fully comprehend prior to ownership. Successful relationships, where owners are satisfied with their dogs, may rely on preconceived expectations being met and therefore our findings suggest a need to instill realistic expectations of dogs, and dog ownership, in aspirant owners to optimise dog and human health and welfare.

## Data availability statement

The datasets presented in this article are not readily available because the data are not publicly available due to consent not collected from participants to share the raw data. Requests to access the datasets should be directed to research@dogstrust.org.uk.

## Ethics statement

The studies involving humans were approved by Dogs Trust Ethical Review Board. The studies were conducted in accordance with the local legislation and institutional requirements. The participants provided their written informed consent to participate in this study.

## Author contributions

KA: Conceptualization, Data curation, Formal analysis, Investigation, Methodology, Project administration, Visualization, Writing – original draft, Writing – review & editing. KH: Conceptualization, Formal analysis, Investigation, Methodology, Writing – original draft, Writing – review & editing. RAC: Conceptualization, Resources, Supervision, Writing – review & editing. BC: Data curation, Formal analysis, Writing – review & editing. RMC: Conceptualization, Data curation, Formal analysis, Investigation, Methodology, Writing – review & editing.
